# Prevalence of normal weight obesity and its associated cardio-metabolic risk factors – Results from the baseline data of the Kerala Diabetes Prevention Program (KDPP)

**DOI:** 10.1371/journal.pone.0237974

**Published:** 2020-08-25

**Authors:** Nitin Kapoor, Mojtaba Lotfaliany, Thirunavukkarasu Sathish, Kavumpurathu R. Thankappan, Nihal Thomas, John Furler, Brian Oldenburg, Robyn J. Tapp

**Affiliations:** 1 Department of Endocrinology, Diabetes and Metabolism, Christian Medical College & Hospital, Vellore, Tamil Nadu, India; 2 Melbourne School of Population and Global Health, Faculty of Medicine, Dentistry and Health Science, The University of Melbourne, Melbourne, Australia; 3 Population Health Research Institute, McMaster University, Hamilton, Ontario, Canada; 4 Centre for Population Health Sciences, Lee Kong Chian School of Medicine, Nanyang Technological University, Singapore, Singapore; 5 Achutha Menon Centre for Health Science Studies, Sree Chitra Tirunal Institute for Medical Sciences and Technology, Trivandrum, Kerala, India; 6 Department of Public Health and Community Medicine, Central University, Kasaragod, Kerala, India; 7 Department of General Practice, Faculty of Medicine, Dentistry and Health Science, The University of Melbourne, Melbourne, Australia; 8 The School of Biomedical Engineering and Imaging Sciences, Kings College London, London, United Kingdom; 9 Centre for Intelligent Healthcare, Faculty of Health and Life Sciences, Coventry University, Coventry, United Kingdom; Kasturba Medical College Mangalore, INDIA

## Abstract

**Background:**

Cardiometabolic disorders are frequently observed among those who have obesity as measured by body mass index (BMI). However, there is limited data available on the cardiometabolic profile of those who are non-obese by BMI but with a high body fat percentage (BFP), a phenotype frequently observed in the Indian population. We examined the prevalence of individuals with normal weight obesity (NWO) and the cardiometabolic profile of NWO individuals at high risk for type 2 diabetes(T2D) in a south Asian population.

**Material and methods:**

In the Kerala Diabetes Prevention Program, individuals aged between 30 to 60 years were screened using the Indian Diabetes Risk Score(IDRS) in 60 rural communities in the Indian state of Kerala. We used data from the baseline survey of this trial for this analysis which included 1147 eligible high diabetes risk individuals(IDRS >60). NWO was defined as BMI within the normal range and a high BFP (as per Asia-pacific ethnicity based cut-off); Non-obese (NO) as normal BMI and BFP and overtly obese (OB) as BMI ≥25 kg/m^2^ irrespective of the BFP. Data on demographic, clinical and biochemical characteristics were collected using standardized questionnaires and protocols. Body fat percentage was assessed using TANITA body composition analyser (model SC330), based on bioelectrical impedance.

**Results:**

The mean age of participants was 47.3 ± 7.5 years and 46% were women. The proportion with NWO was 32% (n = 364; 95% CI: 29.1 to 34.5%), NO was 17% (n = 200) and OB was 51% (n = 583). Among those with NWO, 19.7% had T2D, compared to 18.7% of those who were OB (p value = 0.45) and 8% with NO (p value = 0.003). Among those with NWO, mean systolic and diastolic blood pressure were 129 ± 20; 78 ± 12 mmHg, compared to 127 ± 17; 78±11 mmHg among those with OB (p value = 0.12;0.94) and 120 ± 16; 71±10 mmHg among with NO (p value<0.001; 0.001), respectively. A similar pattern of association was observed for LDL cholesterol and triglycerides. After adjusting for other risk factors, the odds of having diabetes (OR:2.72[95% CI:1.46–5.08]) and dyslipidemia (2.37[1.55–3.64]) was significantly more in individuals with NWO as compared to non-obese individuals.

**Conclusions:**

Almost one-third of this South Asian population, at high risk for T2D, had normal weight obesity. The significantly higher cardiometabolic risk associated with increased adiposity even in lower BMI individuals has important implications for recognition in clinical practice.

## Introduction

Cardiometabolic disorders are one of the leading causes of death worldwide.[1] The conventionally described risk factors including unhealthy dietary patterns and physical inactivity in addition to underlying genetic predisposition are in part responsible for the increasing prevalence of obesity, which is often a forerunner of these cardiometabolic diseases.[2] However, the proportion of people with obesity in India is not as high as in Western countries.[3–5] It has been shown that India has an increasing number of people with diabetes despite a very low prevalence of obesity as measured by body mass index(BMI).[6,7] This disparity may be explained by differences in body composition and distribution of body fat in the otherwise apparently lean Indian phenotype.[8]

Asian Indians have a small body size but often have centripetal obesity as depicted by their high waist-to-hip ratio (WHR), waist circumference, visceral fat and posterior subcutaneous abdominal fat.[9,10] This unique phenotype called normal weight obesity (NWO), is defined as people with normal BMI and high body fat percentage. This phenotype was first described in 1981 by Ruderman et al as metabolically obese normal weight and later termed as normal weight obesity in 2006, by De Lorenzo et al. [11] However, it has not as yet percolated through clinical practice and there remains a rather large proportion of these apparently normal looking individuals with high metabolic risks who go unrecognised in the Indian setting.[5,12]

There is emerging literature on the association of NWO with a high prevalence of cardio-metabolic dysregulation, insulin resistance with associated metabolic syndrome, and other cardiovascular risk factors in western countries.[8,13] In some populations, NWO has also been shown to have an independent association with increased cardio-vascular mortality.[14] However, to our knowledge there are very few studies on the concept of NWO in the Indian population [14–16] and none of them describe the prevalence and the cardio-metabolic risk factors associated with this unique cohort of people.

Therefore, in this study the authors aimed to evaluate the prevalence of NWO and its associated cardio-metabolic risk factors in a south Indian population using baseline data of the KDPP (Kerala Diabetes Prevention Program), a cluster randomised controlled trial of a peer support lifestyle intervention program for the prevention of type 2 diabetes in India.[17]

## Material and methods

The Kerala Diabetes Prevention Program(KDPP) is an ideal study design to evaluate the prevalence of NWO individuals in India. It included standardised anthropometric measurements from individuals recruited from the community, a rigorous assessments of participants’ cardiometabolic risk factors, and a proficient lifestyle based intervention with a sizeable follow-up of individuals at high risk of T2D.[17,18]

KDPP is a cluster-randomised controlled trial of a peer support lifestyle intervention program for the prevention of type 2 diabetes in India. The study design has been described in detail elsewhere.[18] Briefly, individuals from the community between the age of 30 and 60 years with a high diabetes risk (based on Indian Diabetes risk score (IDRS) >60) were recruited into the KDPP study from a random sample of 60 polling areas(electoral divisions) from the Neyyatinkara taluk (sub-district) of Trivandrum district of the Indian state of Kerala. Indian diabetes risk score is a validated risk assessment tool which is used to predict occurrence of diabetes in the Indian population. It uses four risk factors including age of the patient, presence of abdominal obesity, family history of diabetes and the physical activity levels of the individual, to calculate a composite risk score. A risk score of more than 60 is considered to have a high risk of developing T2D.[19] All participants with a IDRS ≥60 were included in this subgroup analysis and those with major chronic illnesses or on medications known to affect glucose metabolism (glucocorticoids, anti-psychotic drugs and anti-retroviral drugs) were excluded.[19] Pregnant women were excluded from the study. All participants were assessed for their socio-demographic characteristics, lifestyle habits and medical history using standardized questionnaires. Anthropometric measurements including height, weight, body fat percent, waist circumference, hip circumference and blood pressure were obtained using standardized instruments and protocols by trained staff.[20] The data collectors were given adequate training on data collection prior to the commencement of the study and refresher training at frequent intervals with a training manual developed in line with the WHO STEPS(Stepwise approach to surveillance) training manual.[21] This refresher training was given three times after the initial training, each before covering 15 electoral divisions(A total of 60 electoral divisions were covered). A more detailed description of the training sessions has been provided in the protocol paper of this study.[17]

Body composition was assessed using TANITA body composition analyser (model SC330), which provided total body fat percentage, based on bioelectrical impedance. Blood samples were collected and centrifuged within 30 minutes and then transported in dry ice to a nationally accredited laboratory. In addition to the OGTT (Oral Glucose Tolerance Test), other biochemical measurements assessed included HbA1c and serum lipids. Plasma glucose was measured using the COBAS 6000 analyser with kits provided by Roche diagnostics utilizing the hexokinase method. HbA1c was measured using the D-10 BIORAD analyser and lipids on a COBAS 6000 analyser by the high-performance liquid chromatography and enzymatic methods, respectively.

Presence of diabetes and prediabetes was defined based on criteria from the American Diabetes Association following a 2-hour 75 gram OGTT. Those with a fasting plasma glucose value ≥ 126 mg/dl and/or 2-h plasma glucose value of ≥ 200 mg/dl were diagnosed to have diabetes. Those with a fasting plasma glucose value between 100 mg/dl and 125 mg/dl with a 2 hour plasma glucose < 140 mg/dl(impaired fasting glucose) and/or a 2 hour plasma glucose between 140 mg/dl and 200 mg/dl with a fasting plasma glucose less than 100 mg/dl(impaired glucose tolerance) were diagnosed to have pre-diabetes. Those with fasting plasma glucose < 100 mg/dl and a 2-h plasma glucose < 140 mg/dl were considered to have normal glucose. [20] Hypertension was defined in those individuals with a systolic BP of ≥ 140 mmHg and/or diastolic BP ≥ 90 mmHg and/or currently taking BP lowering medications. Those with pre-hypertension were defined if they had a systolic BP between 120 and 139 mmHg and/or diastolic BP between 80 and 89 mmHg and were not taking plasma pressure lowering medications.[22] Dyslipidemia was defined as in those individuals who were taking lipid-lowering medications and/or had a high total cholesterol(>200 mg/dl) and/or high LDL cholesterol(>100 mg/dl) and/or low HDL cholesterol(<40 mg/dl in men and <50 mg/dl in women) and/or high triglycerides(>200 mg/dl).[23]

Participants were stratified into three groups based on their body composition and BMI. Individuals with a BMI (≥ 25 kg/m2) were defined as having obesity (OB) and those with a BMI between (18.5–24.9 kg/m2) were further stratified based on their body fat percentage.[24] Normal weight obesity (NWO) was defined as individuals who had a BMI (18.5–24.9 kg/m2) and a high body fat percentage (≥ 20.6% in men and ≥ 33.4% in women) as defined by previously published criteria.[25] Those individuals who has normal body fat (<20.6% in men and < 33.4% in women) and a BMI between 18.5–24.9 kg/m2 were defined as Non Obese(NO) individuals.[26]

The study was approved by the Health Ministry Screening Committee of the Government of India; ethics committees of the Sree Chitra Tirunal Institute for Medical Sciences and Technology (SCT/IEC-333/May 2011), Trivandrum, India; Monash University (CF11/0457-2011000194); and The University of Melbourne (1441736) in Australia. Written informed consent was obtained from all study participants.

Data analysis was performed using stata version 14.0 (StataCorp LP, College Station, TX, USA). Mean ± standard deviation (SD) values are presented for normally distributed variables, and medians (interquartile range) are presented for skewed variables. Categorical variables are summarized with frequencies and percentages. To compare the means of variables between different obesity strata, mixed-effects univariable linear regression models were fitted. The obesity status was considered as the fixed-effect and the study clusters (polling areas) as the random-effect. Skewed variables were log-transformed before analysis. To compare the prevalence of categorical variables between different obesity strata, logistic regressions were fitted using generalized estimating equations with an exchangeable working correlation structure and robust standard errors to account for clustering by polling areas. Results of the generalized estimating equation(GEE) models are presented as odds ratios(OR) and 95% confidence interval [CI], P VALUE.

GEE models were also used to calculate the odds ratio(95% CI and associated p values) for the association between various categories of obesity and diabetes, hypertension and dyslipidemia after adjusting for age, sex, tobacco use and alcohol intake. A two-sided p value <0.05 was considered statistically significant for all analyses.

## Results

Screening and recruitment of individuals was carried out from January to October 2013. A total of 3552 individuals were screened for eligibility of which 1529(43%) individuals who met the inclusion and exclusion criteria were invited for further investigations to a clinic. 1209 individuals attended the clinic of which 62 had a BMI less than 18.5 kg/m^2^ and were excluded as per the obesity definitions. A total of 1147 individuals were included in this analysis.

The mean age of the study participants was 47.3 ± 7.5 years and 46% were women. 50.8% of the participants were found to have overt obesity (50.8%;95% CI: 47.9 to 53.7). Among the 564 individuals with BMI less than 25 kg/m^2^, 364(64.5%; 95% CI: 60.4 to 68.4) were found to have NWO which was 31.7%(95%; CI: 29.1 to 34.5%) of the total study population.

The mean age (±SD) of participants with normal weight obesity was 47.9±7.4 years and was similar to the non-obese 47.2±7.7 years and obese 46.9±7.5 years (p value = 0.06). Table 1 summarizes the comparison of various glycemic parameters among the three groups. It was found that the mean fasting and post 2 hour post glucose parameters of the non-obese group were significantly lower as compared to those with NWO [Mean(SD)—Fasting plasma glucose(mg/dl): NO- 108.1(25.9), NWO-118.5(41.7) (p value = 0.001); 2h post glucose(mg/dl): NO– 112.3(55.9), NWO– 137.4(88.3) (p value = 0.001)]. In addition, significantly more number of individuals were found to have diabetes in the NWO (p value = 0.001) and OB (p value = 0.001) group when compared to NO group [Proportion with diabetes–NO(8%), NWO(19.7%), OB(18.7%)]. As a corollary, the number of non-diabetic individuals were higher in the NO group as compared to the OB (p value = 0.001) and NWO (p value = 0.001) group[Proportion without diabetes–NO(37%), NWO(23.9%), OB(18.8%)]. The remaining of the subjects were in the prediabetes category. There was no statistically significant difference found between the NWO and OB group for mean plasma glucose(Fasting and post prandial) and HbA1c.

**Table 1:** Comparison of glycemic parameters among the different groups of obesity.

Glycemic parameters	Non-Obese(NO) (n = 200)	Normal Weight Obese (NWO) (n = 364)	OBESE (OB) (n = 583)	P value
Mean Fasting Plasma glucose mg/dl (SD)	108.1(25.9)	118.5(41.7)	117.1(34.05)	0.001 (NO vs NWO) 0.002 (NO vs OB) 0.537 (NWO vs OB)
Mean 2 hour plasma glucose mg/dl (SD)*	112.3(55.9)	137.4(88.3)	136.8(68.9)	0.001 (NO vs NWO) 0.001 (NO vs OB) 0.906 (NWO vs OB)
Mean HbA1c % (SD)	5.59(0.58)	5.68(0.74)	5.69(0.87)	0.18 (NO vs NWO) 0.02 (NO vs OB) 0.37 (NWO vs OB)
Participants diagnosed to have diabetes n (%)	16(8)	72(19.78)	110(18.7)	0.001 (NO vs NWO) 0.001 (NO vs OB) 0.717 (NWO vs OB)
Participants diagnosed to have pre-diabetes n (%)	110(55)	205(66)	357(61.2)	0.74 (NO vs NWO) 0.112 (NO vs OB) 0.115 (NWO vs OB)
Participants with normal plasma sugars n (%)	74(37)	87(23.9)	110(18.8)	0.001 (NO vs NWO) 0.001 (NO vs OB) 0.115 (NWO vs OB)

SD- standard deviation

The proportion of participants with hypertension (p value = 0.016) and pre-hypertension (p value = 0.017) in the normal weight obese group were significantly higher than the non-obese group.[Proportion with hypertension–NO (7%), NWO (15.3%), OB (15.4%); proportion with pre hypertension–NO (38.5%), NWO (50.8%), OB (50.7%)] This was also reflected in the mean systolic (p value = 0.001) and diastolic (p value = 0.001) blood pressure values. These parameters were similar among the NWO and OB groups. These values are summarized in Table 2.

**Table 2:** Comparison of blood pressure measurements among the different groups of hypertension.

Blood Pressure	Non-Obese(NO) (200)	Normal Weight Obese (NWO) (364)	OBESE (OB) (583)	P value
Mean Systolic blood pressure mm of Hg (SD)	119.8(16.4)	129.1(19.7)	127.3(17.2)	0.001 (NO vs NWO) 0.001 (NO vs OB) 0.112 (NWO vs OB)
Mean Diastolic blood pressure mm of Hg (SD)	70.8(10.3)	77.5(12.2)	77.8(11.3)	0.001 (NO vs NWO) 0.001 (NO vs OB) 0.945 (NWO vs OB)
Hypertension (n%)	14(7)	56(15.3)	90(15.4)	0.016 (NO vs NWO) 0.004 (NO vs OB) 0.982 (NWO vs OB)
Pre-Hypertension (n%)	77(38.5)	185(50.8)	296(50.7)	0.017 (NO vs NWO) 0.002 (NO vs OB) 0.990 (NWO vs OB)
No Hypertension (n%)	109(54.5)	123(33.7)	197(33.7)	0.001 (NO vs NWO) 0.001 (NO vs OB) 0.997 (NWO vs OB)

SD- standard deviation

The proportion of individuals having dyslipidemia was significantly higher in individuals with normal weight obesity (p value = 0.001) and those with obesity (p value = 0.001) when compared to the non-obese group. [Proportion with dyslipidemia- NO (75%), NWO (89.2%), OB (87.8%)]. The NWO group had a similar proportion of individuals with dyslipidemia as in the OB group (p value = 0.494). The mean total cholesterol (p value = 0.01), serum triglyceride (p value = 0.001) and low density lipoprotein cholesterol (p value = 0.002) was significantly higher among the normal weight obese individuals when compared to the non-obese individuals. The mean HDL cholesterol was significantly lower among the NWO individuals (p value = 0.001) as compared to the NO group. These values are summarized in Table 3.

**Table 3:** Comparison of lipid profile among different groups of obesity.

Lipid Profile	Non-Obese(NO) (200)	Normal Weight Obese (NWO) (364)	OBESE (OB) (583)	P value
Mean Total cholesterol (SD) mg/dl	217.3(38.8)	226.5(42.3)	222.3(39.5)	0.014 (NO vs NWO) 0.152 (NO vs OB) 0.146 (NWO vs OB)
Mean LDL cholesterol (SD) mg/dl	148.3(36.4)	158.6(37)	156.8(35.2)	0.002 (NO vs NWO) 0.006 (NO vs OB) 0.466 (NWO vs OB)
Mean Triglyceride levels (SD) mg/dl	97.3(47.9)	128.9(82.9)	126.2(84.8)	0.001 (NO vs NWO) 0.001 (NO vs OB) 0.563 (NWO vs OB)
Mean HDL cholesterol (SD) mg/dl	55.8(15.5)	49.8(15.1)	47.8(12.6)	0.001 (NO vs NWO) 0.001 (NO vs OB) 0.59 (NWO vs OB)
Dyslipidemia n(%)	150(75%)	325(89.2%)	512(87.8%)	0.001 (NO vs NWO) 0.001 (NO vs OB) 0.494 (NWO vs OB)

SD- standard deviation

On a further subgroup analysis of individuals with normal weight obesity(N = 364) between the overweight (BMI 23.0–24.9 kg/m2, N = 244) and normal weight BMI categories (BMI 18.5–22.9 kg/m2, N = 120), we found no statistical difference between their glycemic, blood pressure and lipid parameters. Following univariate analysis, a multiple regression was fitted to account for age and sex on the association between plasma glucose, blood pressure and serum lipid levels and the three groups of obesities. After adjusting for age and sex, the estimated odds for association did not differ noticeably from those achieved by univariate analysis between any of the parameters. After adjusting for age, sex, tobacco use and alcohol intake the odds of having diabetes(2.72[1.46–5.08]) and dyslipidemia was significantly more in individuals with NWO as compared to non-obese individuals(2.37[1.55–3.64]). (Table 4) This was found to be similar in comparison to the increase in odds obtained in those with obesity vs those who were non obese individuals as detailed in Table 4. Thereby suggesting that the risk of developing T2DM, hypertension and dyslipidaemia was about two-fold higher in individuals with normal weight obesity as compared to non-obese individuals after adjusting for age, sex, tobacco use and alcohol intake.

**Table 4:** Odds of having diabetes, hypertension and dyslipidemia among normal weight obese individuals.

	Normal Weight obesity Vs Non Obese		Obese Vs Non Obese	
	OR[95% CI]*	P. Value	OR[95% CI]*	P. Value
Type 2 Diabetes Mellitus	2.72[1.46–5.08]	0.002	2.75[1.5–5.04]	0.001
Hypertension	1.89[0.92–3.86]	0.082	2.14[1.18–3.87]	0.012
Dyslipidemia	2.37[1.55–3.64]	<0.001	2.44[1.68–3.54]	<0.001

* after adjusting for Age, sex, tobacco use and alcohol intake.

## Discussion

About one third of the study participants in this study were found to have normal weight obesity. There was a significantly higher proportion of individuals with diabetes, hypertension and dyslipidemia in the NWO group as compared to the non-obese group and the plasma glucose, blood pressure and serum lipid levels in the normal weight obese group was similar to the overtly obese group.

This is the first study to analyse the prevalence of NWO in a high diabetes risk Indian population. The prevalence of NWO has been studied in other populations but varies widely based on their ethnicities, the diverse methodologies used for body fat assessment and the different cut-offs points used to define it.[27] Estimates from the United States suggest that there are about 30 million Americans affected with NWO.[14,28] The first study that described NWO, defined it as individuals who had a BMI in the target range (18.5–24.9 kg/m2) but elevated body fat percentage(>30%). In this study, DXA (Dual energy x-ray absorptiometry) was used to measure body fat percentage and they only included women who had no known metabolic disorders. However, in this study the prevalence of NWO was not described.[29] Following this several studies have described the prevalence of NWO in different populations which has ranged from as low as 9% to as high as 34%.[14,15,30,31] In our study we found that about a third of our high diabetes risk study population had NWO and more notably about two-third of those with a non-obese BMI had a high body fat percentage. These figures suggest that there is a significant prevalence of NWO in the Indian population.[8] This could be explained on the basis of the current study being designed to demonstrate the prevalence of NWO in a population at high risk for diabetes and that the Asian Indian phenotype is known to have a higher body fat percentage at a lower body mass index.[32] Nevertheless, a significant proportion of people have this phenotype and need to be identified as a distinctive subset from those with normal weight and normal proportion of body fat.

Excessive body fat, irrespective of BMI is known to be a major risk factor for the evolution of metabolic disorders like diabetes, hypertension and dyslipidemia.[33] In our study, we detected a significantly higher proportion of these disorders in the NWO group when compared to the non-obese group. In another study by Kim et al both men and women with NWO had a higher risk of developing one or more metabolic disorders including diabetes, hypertension and or dyslipidemia (Odds Ratio = 1.63, 95%CI 1.21–2.19 in men;1.56, 95%CI 1.36–1.8 in women) when compared those with appropriate body fat percentage.[34] In addition, the mean fasting and postprandial plasma glucose were also found to be higher in our subjects with NWO. This is similar to another study by Marques et al wherein 3123 women with NWO had a higher odds of developing hyperglycemia when compared to those with normal body fat percentage.(OR = 1.63; 95% CI, 1.10–2.42).[35]

In this study, we found that the mean systolic and diastolic blood pressure was significantly higher in the NWO group when matched to the non-obese group and was similar to the overtly obese group. A comparable finding has been found in other studies among different populations.[14,35,36] In addition to the high prevalence of dyslipidemia, we also found that individually the mean serum total cholesterol, triglyceride and LDL levels were significantly higher and the mean serum HDL level were significantly lower among the NWO group as compared to the non-obese group. In a study by Kang et al participants with NWO had higher mean serum triglyceride levels and lower mean serum HDL levels when compared to individuals with normal BMI and normal body fat percentage.[36] In a yet another study in a Caucasian population Romero-Corral et al reported a proportional relationship between increased body fat percentage in women with an increased risk of dyslipidemia and associated cardiovascular mortality (Hazard ratio = 1.06 for each point increase of body fat percentage: 95%CI, 1.01–1.12).[14]

Apart from individual cardiovascular risk factors, some studies have also shown an increased occurrence of atherosclerosis in individuals with normal weight obesity individuals. In a study comprising of 2078 Koreans with normal BMI and no prior history of coronary artery disease, it was found that subjects with NWO had a higher value of pulse wave velocity and a greater number of soft coronary plaques when compared with those with normal body fat percentage (1474.06 ± 275.4 cm/s vs 1380.76 ± 234.3 cm/s & 21.6% vs 14.5%, respectively). Furthermore, the presence of NWO was suggestive of an independent risk factor for the development of soft coronary plaques (OR:1.46; 95%CI, 1.03–2.07).[37]

In an attempt to explain as to why individuals with this phenotype had a high cardiovascular risk, multiple postulates have been tested by several studies. It has been found that individuals with NWO have higher inflammatory and pro-thrombotic biomarkers such as plasma homocysteine, interleukins, C-reactive protein, and tumour necrosis factor alpha when compared to the non-obese individuals. [12,14,35,36] Furthermore, it has also postulated that hypoxia in adipose tissue, found in people with excess fat results in the over production of reactive oxygen species and activation of kinases inducing over-expression of pro-inflammatory cytokines and subsequent mitochondrial dysfunction in the liver and skeletal tissues.[38] In addition, certain specific genetic polymorphisms are also described more frequently in individuals with NWO which might predispose them to develop metabolic complications.[2,39–42] Moreover the risk of cardiometabolic disorders in this peculiar phenotype may be also explained by altered neonatal programming followed by low birth weight.[43]

The key strength of this study is that, this is the first community-based study looking at the prevalence of normal weight obesity in the South Asian population with rigours cardiometabolic assessment using standardised anthropometric measurements. Limitations of this study include that we used bioelectrical impedance for estimating body fat percentage. Though cited as a limitation here bioelectrical impedance is probably the best available method to assess body fat and is considered a good alternative to DXA Scan, especially in the community setting.[44] This study indicates the prevalence only in a high diabetes risk group and even though a significant proportion of individuals were normal weight obese even in this high risk group, further studies would be needed to evaluate the prevalence in this unique Asian Indian phenotype representing the general population. Moreover, further studies are also needed to understand the impact of lifestyle and therapeutic interventions in people with normal weight obesity.[45]

## Conclusion

Two thirds of apparently normal weight individuals in the KDPP cohort had normal weight obesity and the cardiometabolic risk factors associated with them were significantly greater than the non-obese individuals and were similar to those with obesity. These findings warrant greater awareness among primary care physicians about this entity of normal weight obesity and a paradigm shift in the measurement of obesity from BMI, which is currently the standard of care in most Indian clinics to estimating body fat percentage which will help to identify and manage these high risk individuals.

## Supporting information

S1 File (DO)

S2 File (PDF)

S3 File (XLS)
