# Supplementary material for: Prevalence of normal weight obesity and its associated cardio-metabolic risk factors – Results from the baseline data of the Kerala Diabetes Prevention Program (KDPP)
Source: PLoS One. 2020 Aug 25;15(8):e0237974. doi: 10.1371/journal.pone.0237974 (PMC7446975; doi:10.1371/journal.pone.0237974)
Supplement: S2 File — (PDF) [file pone.0237974.s002.pdf]

Frequencies

```
[DataSet1]
```

Statistics

bmi_cate

N	Valid	364
	Missing	0

bmi_cate

		Frequency	Percent	Valid Percent	Cumulative Percent
Valid	BMI Category 18.5-22.9	120	33.0	33.0	33.0
	BMI Category 23 - 24.9	244	67.0	67.0	100.0
	Total	364	100.0	100.0	

```
T-TEST GROUPS=bmi_cate(0 1)
  /MISSING=ANALYSIS
  /VARIABLES=sysbpmean diabp hba1c totcholmgdl trigsmgdl hdlmgdl ldlmgdl f
pgmgdl twohrpgmgdl
  /CRITERIA=CI(.95).
```

T-Test

```
[DataSet1]
```

Group Statistics

	bmi_cate	N	Mean	Std. Deviation	Std. Error Mean
sysbpmean	BMI Category 18.5-22.9	120	131.94	21.443	1.957
	BMI Category 23 - 24.9	244	127.76	18.725	1.199
diabp	BMI Category 18.5-22.9	120	77.68	13.049	1.191
	BMI Category 23 - 24.9	244	77.51	11.844	.758
hba1c	BMI Category 18.5-22.9	96	5.6968E0	...	...
	BMI Category 23 - 24.9	206	5.6786E0	...	...
totcholmgdl	BMI Category 18.5-22.9	96	227.34	37.724	3.850
	BMI Category 23 - 24.9	206	226.20	44.443	3.097
trigsmgdl	BMI Category 18.5-22.9	96	132.60	70.854	7.231
	BMI Category 23 - 24.9	206	127.20	88.076	6.137
hdlmgdl	BMI Category 18.5-22.9	96	51.55	14.538	1.484
	BMI Category 23 - 24.9	206	49.00	15.465	1.077
ldlmgdl	BMI Category 18.5-22.9	96	157.64	33.663	3.436
	BMI Category 23 - 24.9	206	159.15	38.646	2.693
fpgmgdl	BMI Category 18.5-22.9	120	119.61	39.345	3.592
	BMI Category 23 - 24.9	244	118.09	42.919	2.748
twohrpgmgdl	BMI Category 18.5-22.9	120	140.84	88.827	8.109
	BMI Category 23 - 24.9	244	135.78	88.211	5.647

Independent Samples Test

		Levene's Test for Equality of Variances		t-test for Equality of Means	
		F	Sig.	t	df
sysbpmean	Equal variances assumed	.489	.485	1.907	362
	Equal variances not assumed			1.821	210.500
diabp	Equal variances assumed	.003	.959	.126	362
	Equal variances not assumed			.122	217.479
hba1c	Equal variances assumed	.144	.704	.198	300
	Equal variances not assumed			.184	157.180
totcholmgdl	Equal variances assumed	.168	.682	.218	300
	Equal variances not assumed			.232	215.803
trigsmgdl	Equal variances assumed	.147	.702	.527	300
	Equal variances not assumed			.570	226.623
hdlmgdl	Equal variances assumed	.412	.521	1.358	300
	Equal variances not assumed			1.389	196.318
ldlmgdl	Equal variances assumed	.153	.696	-.330	300
	Equal variances not assumed			-.347	210.700
fpgmgdl	Equal variances assumed	.433	.511	.325	362
	Equal variances not assumed			.335	256.085
twohrpgmgdl	Equal variances assumed	.632	.427	.513	362
	Equal variances not assumed			.512	235.316

Independent Samples Test

		t-test for Equality of Means		
		Sig. (2-tailed)	Mean Difference	Std. Error Difference
sysbpmean	Equal variances assumed	.057	4.179	2.192
	Equal variances not assumed	.070	4.179	2.295
diabp	Equal variances assumed	.900	.172	1.366
	Equal variances not assumed	.903	.172	1.412
hba1c	Equal variances assumed	.843	...	...
	Equal variances not assumed	.854	...	...
totcholmgdl	Equal variances assumed	.827	1.145	5.243
	Equal variances not assumed	.817	1.145	4.941
trigsmgdl	Equal variances assumed	.599	5.405	10.258
	Equal variances not assumed	.569	5.405	9.484
hdlmgdl	Equal variances assumed	.175	2.547	1.876
	Equal variances not assumed	.166	2.547	1.834
ldlmgdl	Equal variances assumed	.742	-1.515	4.590
	Equal variances not assumed	.729	-1.515	4.365
fpgmgdl	Equal variances assumed	.745	1.514	4.658
	Equal variances not assumed	.738	1.514	4.522
twohrpgmgdl	Equal variances assumed	.608	5.059	9.858
	Equal variances not assumed	.609	5.059	9.881

Independent Samples Test

		t-test for Equality of Means	
		95% Confidence Interval of the Difference	
		Lower	Upper
sysbpmean	Equal variances assumed	-.131	8.490
	Equal variances not assumed	-.345	8.704
diabp	Equal variances assumed	-2.514	2.859
	Equal variances not assumed	-2.611	2.955
hba1c	Equal variances assumed	-1.6321502E-1	1.9968346E-1
	Equal variances not assumed	-1.7717631E-1	2.1364475E-1
totcholmgdl	Equal variances assumed	-9.174	11.463
	Equal variances not assumed	-8.594	10.883
trigsmgdl	Equal variances assumed	-14.782	25.592
	Equal variances not assumed	-13.283	24.094
hdlmgdl	Equal variances assumed	-1.144	6.238
	Equal variances not assumed	-1.069	6.164
ldlmgdl	Equal variances assumed	-10.547	7.517
	Equal variances not assumed	-10.120	7.090
fpgmgdl	Equal variances assumed	-7.646	10.675
	Equal variances not assumed	-7.391	10.419
twohrpgmgdl	Equal variances assumed	-14.327	24.445
	Equal variances not assumed	-14.408	24.526

```
CROSSTABS
  /TABLES=hyt diab cholnit BY bmi_cate
  /FORMAT=AVALUE TABLES
  /STATISTICS=CHISQ
  /CELLS=COUNT ROW COLUMN TOTAL
  /COUNT ROUND CELL.
```

Crosstabs

```
[DataSet1]
```

Case Processing Summary

	Cases					
	Valid		Missing		Total	
	N	Percent	N	Percent	N	Percent
hyt * bmi_cate	364	100.0%	0	.0%	364	100.0%
diab * bmi_cate	364	100.0%	0	.0%	364	100.0%
cholnit * bmi_cate	364	100.0%	0	.0%	364	100.0%

hyt * bmi_cate

Crosstab

			bmi_cate		
			BMI Category 18.5-22.9	BMI Category 23 - 24.9	Total
hyt	0	Count	105	203	308
		% within hyt	34.1%	65.9%	100.0%
		% within bmi_cate	87.5%	83.2%	84.6%
		% of Total	28.8%	55.8%	84.6%
	1	Count	15	41	56
		% within hyt	26.8%	73.2%	100.0%
		% within bmi_cate	12.5%	16.8%	15.4%
		% of Total	4.1%	11.3%	15.4%
	Total	Count	120	244	364
		% within hyt	33.0%	67.0%	100.0%
		% within bmi_cate	100.0%	100.0%	100.0%
		% of Total	33.0%	67.0%	100.0%

Chi-Square Tests

	Value	df	Asymp. Sig. (2-sided)	Exact Sig. (2-sided)	Exact Sig. (1-sided)
Pearson Chi-Square	1.144[a]	1	.285		
Continuity Correction[b]	.838	1	.360		
Likelihood Ratio	1.179	1	.278		
Fisher's Exact Test				.354	.181
Linear-by-Linear Association	1.141	1	.285		
N of Valid Cases	364				

a. 0 cells (.0%) have expected count less than 5. The minimum expected count is 18.46.

b. Computed only for a 2x2 table

diab * bmi_cate

Crosstab

			bmi_cate		
			BMI Category 18.5-22.9	BMI Category 23 - 24.9	Total
diab	diabetes	Count	28	44	72
		% within diab	38.9%	61.1%	100.0%

Crosstab

			bmi_cate		
			BMI Category 18.5-22.9	BMI Category 23 - 24.9	Total
diab	diabetes	% within bmi_cate	23.3%	18.0%	19.8%
		% of Total	7.7%	12.1%	19.8%
	no diabetes	Count	27	60	87
		% within diab	31.0%	69.0%	100.0%
		% within bmi_cate	22.5%	24.6%	23.9%
		% of Total	7.4%	16.5%	23.9%
	pre-diabetes	Count	65	140	205
		% within diab	31.7%	68.3%	100.0%
		% within bmi_cate	54.2%	57.4%	56.3%
		% of Total	17.9%	38.5%	56.3%
	Total	Count	120	244	364
		% within diab	33.0%	67.0%	100.0%
		% within bmi_cate	100.0%	100.0%	100.0%
		% of Total	33.0%	67.0%	100.0%

Chi-Square Tests

	Value	df	Asymp. Sig. (2-sided)
Pearson Chi-Square	1.437[a]	2	.488
Likelihood Ratio	1.409	2	.494
N of Valid Cases	364		

a. 0 cells (.0%) have expected count less than 5. The minimum expected count is 23.74.

cholnit * bmi_cate

Crosstab

			bmi_cate		
			BMI Category 18.5-22.9	BMI Category 23 - 24.9	Total
cholnit	absent	Count	9	30	39
		% within cholnit	23.1%	76.9%	100.0%
		% within bmi_cate	7.5%	12.3%	10.7%
		% of Total	2.5%	8.2%	10.7%
	present	Count	111	214	325
		% within cholnit	34.2%	65.8%	100.0%
		% within bmi_cate	92.5%	87.7%	89.3%
		% of Total	30.5%	58.8%	89.3%
	Total	Count	120	244	364
		% within cholnit	33.0%	67.0%	100.0%
		% within bmi_cate	100.0%	100.0%	100.0%
		% of Total	33.0%	67.0%	100.0%

Chi-Square Tests

	Value	df	Asymp. Sig. (2-sided)	Exact Sig. (2-sided)	Exact Sig. (1-sided)
Pearson Chi-Square	1.933[a]	1	.164		
Continuity Correction[b]	1.465	1	.226		
Likelihood Ratio	2.043	1	.153		
Fisher's Exact Test				.207	.111
N of Valid Cases	364				

a. 0 cells (.0%) have expected count less than 5. The minimum expected count is 12.86.

b. Computed only for a 2x2 table
